# Creating effective academic research teams: Two tools borrowed from business practice

**DOI:** 10.1017/cts.2020.553

**Published:** 2020-11-05

**Authors:** Holly H. Brower, Barbara J. Nicklas, Michael A. Nader, Lindsay M. Trost, David P. Miller

**Affiliations:** 1 Clinical and Translational Science Institute, Wake Forest University, Winston-Salem, NC, USA; 2 Clinical and Translational Science Institute (CTSI), Wake Forest School of Medicine, Winston-Salem, NC, USA

**Keywords:** Team development, team charter, RACI matrix, leadership development, team science

## Abstract

Academic Medical Centers strive to create multidisciplinary research teams to produce impactful science. However, few faculty researchers receive training in “team science,” a well-established concept in business research and practice. Responding to demand for assistance developing effective research teams, the Collaboration and Team Science Program of the Clinical and Translational Science Institute (CTSI) at Wake Forest School of Medicine (WFSM) partnered with faculty from the Wake Forest University (WFU) School of Business with expertise in leadership, management, and team building. We initiated a needs assessment, including a written survey from a diverse set of 42 research scientists as well as semi-structured interviews with 8 researchers. In response to identified needs, we developed training sessions and consultations to teach teams to implement two tools known to enhance team dynamics: (1) Team charter, a document that defines the team’s purpose, goals, roles, and strategies; and (2) Responsible, Accountable, Consulted, Informed (RACI) matrix, a table or spreadsheet that clarifies tasks and accountability. Since 2018, 10 teams and over 100 individuals have attended training sessions and 6 teams received personalized team consults. We describe these tools, present a formal analysis of quantitative results, and highlight the next steps being taken in response to these findings.

## Introduction

Complex work of various types, including the design and conduct of high-impact scientific research, hinges on the ability to collaboratively solve problems [1]. This reality has become increasingly evident in medicine and science. Notably, the American Association of Medical Colleges names the ability to “collaborate as a member of an interprofessional team” as one of its 13 core entrustable professional activities for entering residency training [2]. In biomedical research, multidisciplinary teams consistently outperform less diverse research teams [3–5]. If the Clinical and Translational Science Awards (CTSAs) are to meaningfully impact health, they must foster highly productive, diverse clinical and research teams [6]. However, the mere existence of an interdisciplinary team does not guarantee that positive effects will be realized. Best practices for effective collaboration among team members (often referred to as “team science”) must be discovered and implemented within medical care and research teams to foster their success.

The NIH Field Guide on Collaboration and Team Science [7], originally published by the National Cancer Institute in 2010 and updated in 2018, provides resources and tools for effective team operation. Other tools designed to enhance team effectiveness [8, 9] and training curricula [10–12] have also been developed and evaluated. Despite the existence of these tools, few medical school faculties are aware of them or have used them.

Medical schools and graduate research programs have lagged behind in teaching the principles of effective team science [13]. A review of interprofessional primary care teams in health care affirmed that “a general understanding of optimal team design is not available (p. 550)” [14]. Though rigorously trained in science and the conduct of research, few research scientists understand how to effectively develop and work in teams. On the other hand, business school faculty have been investigating and implementing features of highly effective teams for decades in the fields of organizational behavior, management, leadership, and organizational psychology. Recognizing a critical need to train medical school clinical and research faculty in the principles of team science, particularly with an emphasis on translational research, the Wake Forest School of Medicine (WFSM) Clinical and Translational Science Institute (CTSI) reached out to experts in organizational science at our School of Business to develop and implement a novel team science training program for faculty and research staff.

## Approach

In 2017, representatives from the medical school’s CTSI leadership team met with faculty from the Wake Forest University (WFU) School of Business who had expertise in leadership, management, and team building to discuss team science and forge a working partnership. The discussions resulted in two WFU Business School faculty members joining the CTSI’s Collaboration and Team Science Program’s leadership, alongside clinical and basic science researchers, to strategize and guide the development of effective research teams. This paper reports on the development and ongoing work of a team science training program established by this group.

## Needs Assessment

After speaking with CTSI leaders, the experts from the business school recommended several best practices that demonstrate evidence-based success in building high-performing teams across multiple disciplines and applied contexts. They also recommended a number of topics for training and development in areas critical to team formation, cohesion, and effectiveness.

We then sought to prioritize these topics by conducting a needs assessment involving a survey of 42 basic and clinical research faculty accompanied by 8 individual interviews in 2017. Surveyed faculty ranged from early career to established scientists across various disciplines (Table 1). On the survey, participants used a 5-point Likert scale to rate the perceived need for training in nine team science-related competencies identified as critical success factors in high-performing teams. The scale was anchored at 1 = “great need for this training” and 5 = “no need for this training.” The survey also asked respondents to prioritize the nine topics from “1” (highest priority) to “9” (lowest priority). To better understand the survey data and preliminary results, we conducted eight semi-structured interviews following an interview guide with researchers to further explore and understand the specific needs of their research teams. Furthermore, these interviews allowed for a better understanding of the issues that make research teams more cohesive and productive. Survey responses showed, and interviews confirmed, that teams were struggling with the following issues: setting clear expectations; holding each other accountable to both tasks and deadlines; gaining buy-in on goals and objectives from the whole team rather than only senior faculty; and running effective meetings.

**Table 1.** Needs assessment survey results for team science core competencies

Core competency	% of who are listed as a top three priority	Mean rating^*^
Leading effective meetings	54	2.2
Establishing clear, effective expectations	49	2.3
Leveraging conflict	44	2.2
Holding others accountable	44	2.3
Tactics to drive toward results	42	2.3
Making team decisions	33	2.6
Building trust	28	2.7
Effective listening and communication skills	19	2.8
Valuing differences	11	3.2
**Themes from responses to open-ended question: “what is the biggest challenge you face on the teams on which you serve?”** • **Communication** Location is an issue, email complicates our communications, expectations are not clearly communicated, communication about conflicts. • **Expectations** Setting clear expectations of roles, tasks, and deadlines. • **Accountability** Getting members to meet deadlines, set priorities among individual differences and multiple projects, feedback challenges (especially to senior-level team members). • **Engagement** Getting everyone to buy in to goal/vision.		

*n* = 42 research scholars in Medical Center.

* 5-point scale; 1 = great need for training, 5=no need for training.

The needs assessment results and additional input from faculty and CTSI leadership led the Collaboration and Team Science Program to clearly define its mission: *With a goal of producing the most high-impact translational research, this team exists to make the most effective, integrated research teams, then disseminate and share what we*’*ve learned broadly to be thought leaders in the field of team science and contribute to others*’ *excellence as a result.*

## Objectives of the WFSM-WFU Business School Collaboration and Team Science Program

In response to the needs assessment, we developed five primary objectives:

- Facilitate formation of teams within the medical school through promoting research studios and team building/formation of new teams;
- Encourage and facilitate interdisciplinary team science;
- Connect people who wouldn’t normally work together by identifying gaps and bringing people together, as well as facilitating team engagement;
- Cause people to think about their research in a different way;
- Be thought leaders in the field of team science and contribute to others’ excellence as a result.

The focus of this paper is *encouraging and facilitating interdisciplinary team science* using recommendations from well-researched and implemented resources (including the NIH Field Guide). To meet this particular objective, we developed workshops and tools to teach and facilitate effective team formation and norms within research teams. Specifically, these training tools focused on clearly defining a team’s purpose, objectives, roles, developing trust within teams, managing tasks effectively while communicating clear expectations, and delivering effective feedback and conflict management.

We combined the needs assessment results with research on effective teams to develop the training plan and tools that would add the most value to the research teams. The tools and training plan described below were in response to the needs and priorities expressed by respondents.

## Two Specific Tools for Effective Teams

Scientific teams that are carefully formed, nurtured, and developed exhibit better performance [7]. Clarification of roles, clear goal setting, and team cognition are particularly impactful on team performance [1, 15]. Team cognition is the emergent process where teams develop a shared understanding of their overarching purpose and process, as well as what standards of interaction and performance will be expected [4].

We adapted two distinct tools that are having a strong early positive impact on team cognition and resulting effectiveness among our research teams.

1. Team charter: We developed a **team charter** protocol and an accompanying training program for its use (see online Appendix A). A team charter is a document that clearly defines a team’s purpose, goals, strategies, and team members’ roles for holding each other accountable to mutual expectations. Initiated by the team leader, teams agree on clear practices for team processes such as responding to emails, attendance, and timeliness of meetings. Such a team process is an effective way of forming team cognition, “the manner in which knowledge important to team functioning is mentally organized, represented, and distributed within the team and allows team members to anticipate actions, define processes and hold members accountable (p. 33)” [16]. In fact, effective practice mandates that high-performing teams discuss and commit to their mission and purpose, specific roles, goals, and practices and to hold each other accountable [17]. Teams set a charter together with input from every member and affirm their commitment to the charter when it is completed.

Importantly, teams agree on accountability guidelines and expectations about how a conflict will be resolved before there are personalities and specific issues causing division. High-performing teams communicate well, even when there are disagreements because a foundation of trust between members has been established [18]. The process of clearly establishing expectations and procedures builds trust in early formation.

2. RACI matrix: A team charter clearly sets expectations and goals for success, but it often fails to clarify who is responsible for specific tasks, which can be problematic and frustrating for the successful implementation of the team’s goals. We encourage the use of a **Responsible, Accountable, Consulted, Informed (RACI) matrix** to clearly define tasks and responsibilities, allowing teams to effectively track progress and hold members accountable [19]. The RACI matrix is a project management tool (Table 2). It can be utilized to manage team process even without a team charter, but the combination of the two tools is most effective. The team charter sets expectations and roles early in the team formation process, whereas the RACI matrix is a fluid document that is changed and added to throughout the team’s functioning. It is a working document used to manage specific tasks and accountability.

**Table 2.** Example of a RACI ^*^ matrix for a research team

Task	Diana	Anna	Dave	Nicole
Recruit participants	A		I	R
Administer surveys	A	I	C	R
Maintain study databases	R	A	I	
Train clinics on the study protocol	R		C	A
Create an annual progress report	R	C	A	R

* Responsible, Accountable, Consulted, Informed.

Constructing a RACI matrix begins by first listing the team’s specific tasks, such as “administer surveys” or “create annual progress report.” Then, for each task, the team decides who is “Accountable” (the individual ultimately responsible to ensure that the task is completed). To avoid confusion, each task should have only one person labeled “Accountable.” Next, others are assigned tasks according to the roles of “Responsible” (someone who will help do the work, but is not ultimately accountable), “Consulted” (someone who provides input or key information), or “Informed” (someone who is notified of progress but does not contribute to the work) [19]. A RACI matrix clearly defines who “owns” a task (the person responsible for seeing the task through to completion), avoids role confusion, and ensures work is balanced across team members and tasks. Scanning down individual columns allows members to see if a team member is being stretched across too many tasks or being underutilized. If a team member is involved in too many tasks, roles can be downgraded, such as changing a “responsible” to a “consulted.” Scanning across task rows allows members to see if any task is under-resourced. It also provides a simple, clear visual to readily assess progress on each task. As described below, the early impact on teams using the RACI matrix has been remarkable.

### Coaching Teams to use the Tools

Early career faculty: We pilot tested our training materials with the Wake Forest Translational Research Scholars Academy, an institutionally sponsored career development program for early career faculty researchers. Three different 75-minute seminars on team science were added to the curriculum beginning in the Fall of 2017. The seminars involved sharing information and team science best practices, working through case studies, and discussions. The first two seminars, “Establishing Clear Expectations and a Successful Work Plan,” and “Holding Teams Accountable and Addressing Conflict” revolved around developing and using a team charter. The third seminar, “Effective Team Meetings,” was on principles of effective meetings, including pre-work and follow-up after meetings. Examples of these effective meeting principles include sending an agenda prior to meetings, planning specific timing for discussion topics to manage time carefully, and sending meeting minutes/notes within 1 week after a meeting with highlighted action steps.

Broader research audiences: After testing the content with early career faculty, we next implemented a process to share the information more broadly. As a result, two “Lunch-and-Learn” seminars were offered to all faculty and staff within the Medical Center in 2018. These seminars, attended by approximately 105 individuals, focused on principles and practices of effective meetings and trust-building. In addition, we conducted two half-day workshops for research teams across the Medical Center to understand and develop team charters; three months later, a follow-up 2-hour workshop was held for the continued development of their team charters. These workshops were very practical and action-oriented. Participants attended with their specific research teams. Throughout the workshops, teams engaged in exercises and discussions to build the foundation of effective teams according to a model prescribed by Patrick Lencioni [18]. Participation in these workshops provided opportunities for teams to bond with each other, while being coached through the process of developing their team charters. The result was that teams left the workshops with a draft of their team charter.

In addition to seminars and workshops, our Collaboration and Team Science Program offered a consultation with WFU Business School faculty experts for research teams in the Medical Center. Of the six individual team consults held in 2018–2019, three involved assisting additional teams in developing their team charters and one involved an already chartered team in implementing a RACI matrix to effectively manage tasks and expectations. Two other consults involved leadership coaching and conflict resolution.

## Outcomes

1. Team charters: To date, 10 teams consisting of 71 individuals have been trained to implement team charters through workshops. We received 32 evaluations from attendees of team charter training sessions. Both early career faculty (*n* = 15) and senior faculty or executive administrative staff (*n* = 17) rated the training sessions highly favorably. On a scale of 1 (poor) to 5 (excellent), the mean ratings of the sessions were 4.5 for early career faculty and 4.6 for senior faculty/staff. Similarly, both groups reported they would recommend the session to a colleague (mean rating 4.5 for early career faculty and 4.3 for senior faculty/staff). The early career faculty completed a lengthier evaluation with additional items, and 93% (14/15) agreed the content was relevant to working in research teams, 93% (14/15) reported they acquired new skills, and 87% (13/15) reported they were confident applying the new skills acquired.

2. RACI matrix: We collected qualitative feedback from two teams that are using the RACI matrix. Project managers reported that the tool has helped “balance workload among faculty and staff, … enabled us to determine where additional effort was needed …, and helped give the leadership peace of mind knowing that most, if not all, areas of responsibility were covered.” Additionally, the RACI matrix “prevents confusion in regards to responsibility and is a great onboarding tool that helps new members quickly understand roles on the team.”

## Next Steps

Early results show that research teams are responding well to training and the use of tools for facilitating team effectiveness/organization. While our initial evaluation has relied on self-assessment, we recognize the limitations of this method and are implementing more performance-based evaluation plans. These include tracking of team performance before and after receiving a Team Science Consultation, creating a team charter, or implementing RACI. We are also tracking the institution-wide utilization of these tools. We are receiving increasing numbers of requests for consults and training in these tools and other skills related to team leadership.

Our Collaboration and Team Science Program aspires to have every team utilize the team charter process (as required in many business settings) whenever a project team is formed. The RACI matrix becomes the operating tool to implement the agreed-upon purpose, goals, roles, and expectations outlined in the team charter, so the combination of the two tools are particularly effective at producing high-impact teams. We believe the small number of teams using the RACI tool to date is primarily due to the early stage of our work in this area. We are promoting the use of RACI matrices during team consults, and we have anecdotal evidence that additional teams have adopted this practice, as well. We expect the use to become a common practice as our number of team consults increases and trained team members spread across additional teams. An important future metric will be whether these training tools enhance multidisciplinary, translational research success as reflected in funding and high-impact publications.

We also aim to train team leaders to effectively facilitate team process excellence so they are more equipped to independently lead and manage successful teams. To that end, we launched a Leadership Academy early in 2020 for research leaders. This leadership development program is comprised of five 2-hour interactive training sessions on trust building, conflict resolution and feedback, delegation and team management, decision-making, and self-awareness and development. Due to COVID-19 social distancing mandates, we finished our first Leadership Academy via video conference, which was well received. Applications for the second iteration of the Leadership Academy will be received late in Fall 2020.

Finally, part of the mission of the Wake Forest School of Medicine CTSI is to spread the use of these effective tools to other Academic Medical Centers. The research literature and our initial results provide clear evidence that these practices enhance team outcomes, including productivity, communication, satisfaction, and longevity. Therefore, we plan to continue to carefully evaluate and disseminate our findings in future manuscripts and conferences.

## Supplementary material

For supplementary material accompanying this paper visit https://doi.org/10.1017/cts.2020.553. click here to view supplementary material

## References

[1] DeChurch LA , Mesmer-Magnus JR . The cognitive underpinnings of effective teamwork: a meta-analysis. Journal of Applied Psychology 2010; 95: 32–53. 10.1037/a0017328 20085405

[2] Association of American Medical Colleges [Internet]. Core Entrustable Professional Activities for Entering Residency Faculty and Learners’ Guide, 2014. (https://store.aamc.org/downloadable/download/sample/sample_id/66/%20)

[3] Hall KL , Stokols D , Stipelman BA , *et al.* Assessing the value of team science: a study comparing center- and investigator-initiated grants. American Journal of Preventive Medicine 2012; 42: 157–163. 22261212 10.1016/j.amepre.2011.10.011 PMC3586819

[4] Wuchty S , Jones BF , Uzzi B . The increasing dominance of teams in the production of knowledge. Science 2007; 316: 1036–1038. 17431139 10.1126/science.1136099

[5] Hall KL , *et al.* The science of team science: a review of the empirical evidence and research gaps on collaboration in science. American Psychologist 2018; 73: 532–548. 10.1037/amp0000319 29792466

[6] Selker HP , Wilkins CH . From community engagement, to community-engaged research, to broadly engaged team science. Journal of Clinical and Translational Science 2017; 1: 5–6. 10.1017/cts.2017.1 PMC6798217 31660208

[7] Bennett LM , Gadlin H , Marchand C . Collaboration and Team Science Field Guide. 2nd ed. Bethesda, MA: National Institutes of Health Publication No. 18–7660, National Cancer Institute, 2018.

[8] Bennett LM , Maraia R , Gadlin H . The ‘Welcome Letter’: a useful tool for laboratories and teams. Journal of Translational Medicine & Epidemiology 2014; 2(2): 1035. 28649581 PMC5479682

[9] Nancarrow S , Smith T , Ariss S , Enderby PM . Qualitative evaluation of the implementation of the Interdisciplinary Management Tool: a reflective tool to enhance interdisciplinary teamwork using structured, facilitated action research for implementation. Health & Social Care in the Community 2015; 23(4): 437–448. 25522769 10.1111/hsc.12173

[10] Depp CA , Howland A , Dumbauld J , Fontanesi J , Firestein D , Firestein GS . Development of a game-based learning tool for applied team science communication in a virtual clinical trial. Journal of Clinical Translational Science 2018; 2(3): 169–172. 30370069 10.1017/cts.2018.8 PMC6199551

[11] Spring B , *et al.* Online, cross-disciplinary team science training for health and medical professionals: evaluation of COALESCE (teamscience.net). Journal of Clinical Translational Science 2019; 3(2–3): 82–89. 31660230 10.1017/cts.2019.383 PMC6802413

[12] Mayowski CA , Norman MK , Schenker Y , Proulx CN , Kapoor WN . Developng a team science workshop for early-career investigators. Journal of Clinical Translational Science 2019; 3(4): 184–189. 10.1017/cts.2019.391 PMC6799325 31660242

[13] Begg MD , *et al.* Graduate education for the future: new models and methods for the clinical and translational workforce. Clinical and Translational Science 2015; 8: 787–792. 26643714 10.1111/cts.12359 PMC4709034

[14] Wranik WD , *et al.* Implications of interprofessional primary care team characteristics for health services and patient health outcomes: a systematic review with narrative synthesis. Health Policy 2019; 123: 550–563. 30955711 10.1016/j.healthpol.2019.03.015

[15] Klein C , *et al.* Does team building work? Small Group Research 2009; 40: 181–222.

[16] Kozlowski SWJ , Ilgen DR . Enhancing the effectiveness of work groups and teams. Psychological Science in the Public Interest 2006; 7: 77–124. 26158912 10.1111/j.1529-1006.2006.00030.x

[17] Society for Human Resource Management [Internet]. Developing and Sustaining High-Performance Work Teams, 2015. (https://www.shrm.org/resourcesandtools/tools-and-samples/toolkits/pages/developingandsustaininghigh-performanceworkteams.aspx)

[18] Lencioni P . The Five Dysfunctions of a Team. San Francisco, CA: Jossey-Bass, 2002.

[19] CIO [Internet]. *The RACI Matrix: Your Blueprint For Project Success*, 2018. (https://www.cio.com/article/2395825/project-management-how-to-design-a-successful-raci-project-plan.html)

