# Supplementary material for: Creating effective academic research teams: Two tools borrowed from business practice
Source: J Clin Transl Sci. 2020 Nov 5;5(1):e74. doi: 10.1017/cts.2020.553 (PMC8057410; doi:10.1017/cts.2020.553)
Supplement: Supplementary file 1 [file S2059866120005531sup001.docx]

**Supplemental Online Appendix A: Blank Team Charter Protocol**

**Team Charter**

Your team charter template is divided into three major sections:

1. Identifying expectations
2. Establishing team procedures and standards to which team members will be held accountable
3. Specifying the consequences for failing to follow these procedures and fulfill these expectations

The purpose of the team charter is to: accelerate your team's development, clarify roles and expectations, increase individual accountability for team tasks, and reduce the possibility for detrimental team conflict. As such, make your charter **as specific as possible**: (a) specify each task as detailed as possible, (b) specify each step in a procedure or process as detailed as possible, (c) specify the exact person(s) responsible for each task, and (d) specify the exact time and exact place for completion or submission of each task. The more specifically you describe your team expectations, roles, and procedures, the greater chance you have for a successful team experience.

You do not have to follow a specific format, but you want to make the charter useful and active, so keep it short and readable with direct, clear language. Each member of the team should concur and sign a copy of your finalized charter.

Once your team charter has been developed, your team is ready to begin work on collaborative assignments. However, you may soon find that your team is not working as well as you had hoped. This is normal but needs to be attended to immediately. Perhaps your team is simply not following the established charter procedures or roles as strictly as they should be, or perhaps you need to change some of the procedures or roles as outlined in your charter. Call a team meeting immediately to discuss and resolve the challenges your team is facing; do not delay.

**TEAM CHARTER for XX (agree on a name)**

**Team Members:**

*List team member names and contact information.*

| **Team Context and Expectations** |
| --- |

**Context**

*In this section, the team establishes clear, practical, concrete guidelines on*

- *A core purpose*
- *Core values*
- *Goals*
- *Roles and Responsibilities*

**Mission and Objectives**

*Many team members will fall into the trap of assuming that what they personally expect, hope and plan for will be the same for everyone on the team. Talking through candidly each member’s expectations for outcomes of the team is important to the clarity and commitment to overall team outcomes. Possible topics to discuss might include the quality and type of scholarly works produced, skills acquisition, and the development of future grant applications.*

**Composition and Roles**

*Make sure that each member of the team has a meaningful role and clear expectations about activity level and involvement with the team.*

*Examples of roles might include:*

- ***Meeting Facilitator*** *(responsible for collecting information and creating meeting agenda, attending each team meeting and ensuring that group stays on task and follows the agenda);*
- ***Devil’s Advocate*** *(responsible for raising differing points of view and encouraging others to do so);*
- ***Scribe/Recorder*** *(responsible for capturing notes and conclusions from each meeting and publishing them for the team’s reference);*
- ***Editor*** *(responsible for final revisions of documents created by the team);*
- ***Communications Coordinator*** *(responsible for scheduling team meetings and locations and communicating important information between meetings.)*

*These roles can rotate or be assigned to an individual, with specific expectations regarding meeting attendance and involvement identified.* ***Even for the research roles of Principal Investigator and Co-Investigator, teams will benefit from a very specific set of expectations of contributions expected from each member.***

| **Team Procedures** |
| --- |

*In this section, the team identifies clear expectations for*

- *Meeting times*
- *Acceptable behavior during meetings (i.e. laptop use, email, phones, and so on)*
- *Timeliness for responding to one another and in what medium*
- *Delivery of expected tasks by individual team members, including time sensitivity and thoroughness of completion*
- *Outcome measures*

1. Day, time, and place for regular **team meetings**:
2. Preferred method(s) of **communication** (e.g., e-mail, cell phone, wired phone, webex, face-to-face, in a certain meeting room/lab) in order to inform each other of team meetings, announcement, updates, reminders, problems:
3. **Decision-making policy** (by consensus? by majority vote?):
4. Method for setting and following meeting **agendas** (Who will set each agenda? When? How will team members be notified/reminded? Who will be responsible for the team following the agenda during a team meeting? What will be done to keep the team on track during a meeting?):
5. Method of **record keeping** (Who will be responsible for recording & disseminating minutes? How & when will the minutes be disseminated? Where will all agendas & minutes be kept?):

**Work Quality**

1. Project standards (What is a realistic level of quality for team presentations, collaborative writing, individual research, preparation of drafts, peer reviews, etc.?):
2. Strategies to fulfill these standards:

**Team Participation**

**Task Focused**

1. Strategies to ensure cooperation, equal distribution of tasks, and task progress:

**Relationship Focused**

1. Strategies for encouraging/including ideas from all team members, and respect for differing needs and perspectives:
2. Strategies for keeping on task (task maintenance):
3. Preferences for leadership (informal, formal, individual, shared):

**Personal Accountability**

1. Expected individual attendance, punctuality, and participation at all team meetings:
2. Expected level of responsibility for fulfilling team assignments, timelines, and deadlines:
3. Expected level of communication with other team members:
4. Expected level of commitment to team decisions and tasks.

| **Consequences for Failing to Follow Procedures and Fulfill Expectations** |
| --- |

1. Describe, as a group, how you would handle **infractions** of any of the obligations of this team contract:
2. Describe what your team will do **if the infractions continue**:

******************************************************************************

1. *I participated in formulating the standards, roles, and procedures as stated in this charter.*
2. *I am committed to abiding by these terms and conditions.*
3. *I understand that if I do not abide by these terms and conditions, I will suffer the consequences as stated in this charter.*

1) ___________________________________________________date_______________

2) ___________________________________________________date_______________

3) ___________________________________________________date_______________

4) ___________________________________________________date_______________

5) ___________________________________________________date_______________
